# Targeting the Redox Balance in Inflammatory Skin Conditions

**DOI:** 10.3390/ijms14059126

**Published:** 2013-04-26

**Authors:** Frank A. D. T. G. Wagener, Carine E. Carels, Ditte M. S. Lundvig

**Affiliations:** Department of Orthodontics and Craniofacial Biology, Nijmegen Centre for Molecular Life Sciences, Radboud University Nijmegen Medical Centre, PO Box 9101, 6500 HB Nijmegen, The Netherlands; E-Mail: c.carels@dent.umcn.nl

**Keywords:** ROS, oxidative stress, inflammation, antioxidant, skin

## Abstract

Reactive oxygen species (ROS) can be both beneficial and deleterious. Under normal physiological conditions, ROS production is tightly regulated, and ROS participate in both pathogen defense and cellular signaling. However, insufficient ROS detoxification or ROS overproduction generates oxidative stress, resulting in cellular damage. Oxidative stress has been linked to various inflammatory diseases. Inflammation is an essential response in the protection against injurious insults and thus important at the onset of wound healing. However, hampered resolution of inflammation can result in a chronic, exaggerated response with additional tissue damage. In the pathogenesis of several inflammatory skin conditions, e.g., sunburn and psoriasis, inflammatory-mediated tissue damage is central. The prolonged release of excess ROS in the skin can aggravate inflammatory injury and promote chronic inflammation. The cellular redox balance is therefore tightly regulated by several (enzymatic) antioxidants and pro-oxidants; however, in case of chronic inflammation, the antioxidant system may be depleted, and prolonged oxidative stress occurs. Due to the central role of ROS in inflammatory pathologies, restoring the redox balance forms an innovative therapeutic target in the development of new strategies for treating inflammatory skin conditions. Nevertheless, the clinical use of antioxidant-related therapies is still in its infancy.

## 1. Introduction

The primary function of healthy skin is to form a physical and chemical barrier between the external environment and the organism’s internal milieu to defend against injurious insults. Harmful stimuli such as trauma, pathogens or irritants evoke a complex response known as inflammation (1). Inflammation protects organisms against pathogenic invaders and cleans up damaged cells after injury to prevent further tissue damage. Hereto the injurious agents are identified and eliminated and wound healing is initiated for re-establishment of tissue homeostasis. The strength and duration of the inflammatory response depends on stimulus and context, however, and the initial steps are stereotyped as part of the innate immune response [1]. The five classical signs of acute inflammation are: pain, heat, redness, swelling, and functional loss. These signs can be explained by the different phases that the inflammatory response generally follows: (1) dilation of capillaries to increase blood flow; (2) vasopermeabilization; (3) leukocyte recruitment; (4) elimination of pathogens or injurious stimuli; and 5) resolution of inflammation. Unfortunately, inflammation can also become chronic and destructive, and accumulating evidence demonstrate a contribution of chronic inflammation to the development of diseases like Alzheimer’s disease, atherosclerosis, and type 2 diabetes [2].

In most cases, inflammation of skin represents a beneficial and protective process after injury or infection [3]. However, the skin can also be subjected to excessive inflammatory responses resulting in chronic inflammation, auto-inflammation and auto-immunity [4]. The epidermal layer of the skin is composed of predominantly keratinocytes, a few Langerhans cells—which are specialized dendritic cells—and some pigment-producing melanocytes [3,4]. Keratinocytes produce different keratins that generate the toughness of the epidermis [5]. Embedded in the connective tissue of the underlying dermis different types of immune cells can be found, such as macrophages, T cells and mast cells [4]. The connective tissue is composed of an extracellular matrix produced and secreted by fibroblasts, the principal cell type of the dermis [6].

Under homeostatic conditions, the skin surface is colonized by a diversity of microorganisms. However, a dynamic, healthy equilibrium between the epidermis and the microorganismal population is regulated by production of antibiotic and antifungal compounds by dermal sebocytes as well as the microorganisms themselves. Additionally, keratinocytes also produce antibacterial substances constitutively and after infection or injury [7]. Some of these keratinocyte-derived antimicrobics and cytokines influence the immunological properties of dendritic cells and T cells [3,4]. Thus, the skin balances between ensuring an efficient pathogen defense and immunosurveillance and to reduce excessive immune responses that can lead to disease [1].

At the molecular level, inflammation is activated by the inflammasome that is a cytosolic multiprotein complex regulating caspase-1 activation. Caspase-1 subsequently activates the pro-inflammatory cytokines IL-1β and IL-18 by proteolytic cleavage. The inflammasome is composed of the danger sensor protein NALP and the caspase-1 recruiter protein ASC [8]. Dependent on the activation stimuli, different inflammasomes are formed as a response to danger signals as each inflammasome has unique roles in pathogen recognition [8].

Signal transduction by IL-1β and IL-18 results in a series of phosphorylation and ubiquitination events that ultimately leads to activation of nuclear factor (NF)-κB and p38 mitogen-activated protein kinase (MAPK) pathways, which cooperatively induce the expression of IL-1 target genes, including IL-6 [9]. Pathogen-mediated activation of the inflammasome also induces a specific form of caspase-1-dependent cell death, pyroptosis, that results in osmotic swelling and plasma membrane rupture [10,11]. This induces a strong inflammatory response via the release of pro-inflammatory cytokines and spreading of pro-inflammatory molecules [12].

In contrast to immune cells, human keratinocytes constitutively express inflammasome proteins and are a potent source of the pro-inflammatory cytokines pro-IL1α and pro-IL1β [13–15], which are activated and released upon UV exposure [13,16]. This implies important roles for keratinocytes as non-professional immune cells following sunburn, and in innate immune responses dependent on the inflammasome and IL1β. Keratinocytes are thus important in the inflammatory response under both physiological and pathological conditions [13,17].

Notably, it has been reported that the NLRP3 inflammasome assembly is stimulated in a ROS-sensitive manner by ROS generation [18,19]. The predominant source of ROS as response to danger stimuli are mitochondria, which also control inflammation via release of mitochondrial DNA [20,21]. Excessive release of ROS by mitochondria, activated leukocytes and endothelial cells in chronic inflammatory conditions can ultimately result in severe cell and tissue damage and can further promote and aggravate inflammatory injury. In many inflammatory diseases, currently available intervention strategies fail or are of limited success, warranting the need for novel strategies to treat chronic inflammatory conditions. The prolonged presence of oxidative stress is postulated to promote and fuel these deleterious inflammatory processes and may form a novel target for treatment of chronic inflammatory conditions.

## 2. Reactive Species Mediate Cellular Signaling

ROS are free radicals generated from molecular oxygen, such as superoxide anion (O_2_^•−^), hydroxyl radical (HO•), and non-radical species including hydrogen peroxide (H_2_O_2_). Today, it is well accepted that ROS mediate and modulate signaling processes [22,23]. In moderate concentrations, ROS induce a cascade of cell signaling networks, triggering a ROS wave that propagates throughout tissues carrying signals across large distances [24]. They thereby act as important regulatory mediators in different signaling pathways and processes, including cell proliferation, differentiation, and apoptosis [25,26].

Today H_2_O_2_ is recognized as a major ROS signaling molecule [27] that can mediate oxidation of protein thiols [28], and it is widely accepted that ROS can elicit cellular effects by covalently modifying amino acids and subsequently affecting protein activity. Redox-regulated proteins sense the changes in cellular redox state by different mechanisms. Several transcription factors, including nuclear factor κB (NF-κB), hypoxia inducible factor-1 (HIF-1), and p53, contain redox-sensitive cysteine residues in their DNA binding sites [29]. Oxidative modifications of these residues affect DNA binding and subsequently regulate gene transcription of redox sensitive genes [26,30,31]. Additionally, the DNA binding properties of these transcriptional regulators are further indirectly affected by redox-sensitive proteins, like apurinic/apyrimidinic endonuclease/redox effector factor-1 (APE/Ref-1) protein and histone deacetylases [32,33].

Oxidative posttranslational modifications form a major redox-regulated mechanism of protein function [34], targeting multiple types of amino acids with various susceptibilities and subsequent structural and functional consequences [35]. For instance, reversible oxidation of cysteines and oxidative nitration of tyrosines regulate the activity of various kinases involved in signaling pathways, including c-Jun N-terminal kinases (JNK), mitogen-activated protein kinase (MAPK), and protein kinase C [26]. Altered kinase activities due to oxidative modifications affect downstream signaling pathways and consequently transcription factor activation and contribute to additional redox-regulated gene transcription [36]. Effects of some of these redox-mediated alterations of signaling are exemplified below.

Based on the chemical characteristics, only a few amino acids can undergo oxidative modification, namely cysteine, methionine, and tyrosine [37]. Selective oxidation, reduction or chemical modification of these sensor thiols results in a change in protein activity and signal transduction in response to redox fluctuations [38]. However, the reactivity and modification of a particular cysteine residue is highly dependent on the microenvironment [28,39].

The side chain of a cysteine residue contains a terminal thiol (–SH) as a functional group allowing multiple oxidation states and is a common event in redox signaling [27,40–43]. Oxidation of these residues result in reactive sulfenic acid (–SOH) that can form disulfide bonds (RS–SR’) with nearby cysteines or undergo further oxidation to sulfinic acid (–SO_2_H) and sulfonic acid (–SO_3_H) [44]. Most of these modifications are generated by diffusible small molecules like H_2_O_2_ and are reversible by reducing systems like thioredoxin and peroxiredoxin with the exception of the sulfinic and sulfonic states [27]. For instance, reversible inhibition of protein tyrosine phosphatase (PTP) activity and subsequently regulation of cellular tyrosine phosphorylation and downstream signaling is mediated by H_2_O_2_-mediated oxidation of the catalytic cysteine to the sulfenic form [45,46].

Disulfide bond formation is important for protein structure and function [47], and recently its role as signaling event has been demonstrated [48,49]. Some cysteine-containing proteins also form intra- and intermolecular disulfide bonds, resulting in conformational changes and altered function due to ROS-mediated oxidation. For instance, ROS-mediated cysteine oxidation of PTPs is accompanied by intramolecular disulfide bond formation to protect against further oxidation [50–53]. Moreover, the accompanying conformational change might ease access for reducing enzymes and subsequent reactivation of the enzyme [54]. Cysteine redox switches in enzymes have been extensively discussed elsewhere [55].

*S*-glutathionylation is one of the most common S-thionylation reactions inside the cell and is generated from the reaction between cysteine sulfenic acid and a reduced thiol like GSH. This forms a mixed glutathione disulfide (GSSG) that prevents further irreversible oxidation [56]. Hereby, cells can effectively and reversibly respond to redox input, and glutathionylation indeed regulates most cellular pathways [30]. For instance, activation of the master regulator of several antioxidant genes, NF-E2-related factor 2 (Nrf2), is physically confined to binding partner Keap1 in the cytosol [57]. Keap1 glutathionylation leads to dissociation of the Nrf2-Keap1 complex, allowing Nrf2 to translocate to the nucleus and activate expression of target genes [58]. S-glutathionylation has also been found to control NF-κB pathway activation on different levels [30]. Several members of this pathway are inhibited by *S*-glutathionylation [59], and NF-κB itself is subjected to redox-regulation [36,60]. Oxidative modifications may also modulate protein–protein interactions and thereby affect the stability of protein complexes and activity of the protein partners involved [61]. For instance, NF-κB is sequestered by IκB in the cytosol. ROS activate IκB kinases that phosphorylate IκB, leading to its degradation by the proteasome and exposure of the nuclear localization sequence of NF-κB. Next, NF-κB translocates to the nucleus, and activates transcription of various target genes [62]. Moderate ROS levels promote NF-κB activation and cell survival, whereas high levels of ROS inactivate NF-κB, leading to cell death [26,36].

*S*-nitrosylation is the nonenzymatic adduction of NO to a thiol group (-SNO), and is reversible through the action of the protein denitrosylation system mediated by reduced glutathione (GSH) or the thioredoxin system [63,64]. Redox effects of NO are mediated by S-nitrosylation [65] that is thought to exert its regulatory effects through direct modification of protein function via conformational changes or through the protection of thiol groups against further oxidation [66]. However, the exact mechanisms underlying S-nitrosylation-mediated regulation is still not fully explored. S-nitrosylation plays a dual role as it can act as a protective modification and as an intermediate to further oxidation [67] as well as intermediate for formation of secondary posttranslational modifications such as ubiquitination [68]. Also, *S*-nitrosylation mediates the anti-inflammatory effects of NO in the cardiovascular system, as *S*-nitrosylation of *N*-ethylmaleimide factor suppresses vascular inflammation as well as inhibits NF-κB-dependent expression of pro-inflammatory cytokines and adhesion molecules [65].

Mitochondrial ROS production is also a participant in redox signaling networks [69,70]. The respiratory chain generates O_2_^•−^ that is converted to H_2_O_2_, and both species can act as redox signals, but with different properties and interactions [27,71]. Membrane-impermeable O_2_^•−^ is restricted to the mitochondrial matrix where it is thought to act as a redox signal within the mitochondria [72,73]. O_2_^•−^ itself is not particularly reactive with most biomolecules except for iron–sulfur proteins, NO, and quinones [74–76]. Mitochondrial aconitase is an Fe-S cluster containing protein that reacts with O_2_^•−^ to release H_2_O_2_ and ferrous iron [77]. ROS-mediated damage to the Fe–S cluster leads to aconitase inactivation and a cellular metabolic shift from ATP production to fat storage due to coenzyme A buildup [78]. This feedback loop has been suggested to be an antioxidant defense mechanism by reducing respiration and subsequently mitochondrial ROS formation [72,78]. Indeed, mitochondrial oxidative stress has been suspected to contribute to metabolic syndrome [78].

Also, mitochondrial ROS production is thought to play a role in oxygen sensing via HIF-1 [79,80] under both hypoxic [81] and non-hypoxic conditions [82]. Additionally, NADPH oxidases contribute for ROS formation and redox-dependent HIF-1 stabilization [83]. HIF-1 is a heterodimeric transcription factor that activates numerous genes involved in hypoxic adaptation via its binding to a hypoxia response element (HRE) in gene promoter regions [84]. Under normoxic conditions, the HIF1α subunit is tagged at the oxygen-dependent degradation domain for ubiquitination and subsequent proteasomal degradation by oxygen- and iron-dependent prolyl hydroxylases. Under hypoxia, the hydroxylation of HIF-1α subunit is inhibited, thereby enabling the formation of the active HIF transcription factor and subsequent induction of target gene transcription [79]. This is achieved through ROS-mediated oxidation of ferrous iron that is a co-activator of a prolyl hydroxylase function [85]. Moreover, a recent study describes perinuclear accumulation of mitochondria due to an altered microtubule-dependent transport under hypoxia [86]. As a result, increased levels of ROS in the nucleus caused oxidative modifications of DNA bases in the HRE of the VEGF promoter that is important for facilitated assembly of the HIF-1 transcriptional complex and thus increased VEGF gene expression [80,86].

Together, these examples illustrate that ROS-mediated posttranslational modifications act as key regulatory mediators in different signaling pathways and processes, including cell proliferation, differentiation, and apoptosis [25,26]. Paradoxically, ROS-signaling also promotes pathways protecting against oxidative stress to restore an imbalanced ratio between cellular oxidants and antioxidants [87]. Also, from these examples it becomes clear that the redox system affects protein function and biological activity either indirectly at the level of protein expression or stability or directly through posttranslational modifications [26]. An exhaustive collection of literature further points towards the importance of well-regulated ROS-mediated signaling as ROS has been linked to diverse groups of diseases, ranging from cardiovascular problems to neurodegeneration, see e.g., [88]. However, despite the ever-increasing number of studies, it is evident that ROS-mediated signaling and importantly, the regulation and specificity hereof, is still poorly defined and warrants for more investigation [27].

## 3. ROS and (Chronic) Inflammation of the Skin

Excess levels of ROS due to overproduction or because of insufficient scavenging generate oxidative stress, leading to injurious effects via: (1) oxidative modification and damage of biomolecules, altering lipid/protein/DNA structure and function; (2) further irreversible oxidation of reactive protein thiol groups which is hallmark of oxidative stress [89]; and (3) dysregulation of cell signaling pathways [90], triggering downstream signaling cascades leading to altered cytokine release and exacerbation of inflammation [91]. Combined, excess ROS lead to pathological changes in cells and tissues, as exemplified by inflammatory skin conditions like psoriasis.

Inflammatory skin diseases range from acute rashes with itching and redness to chronic conditions like dermatitis (eczema) and psoriasis. Acute skin inflammation may develop following exposure to, e.g., UV radiation (sunburns), allergens, physical wounding, or contact with chemical irritants, and is resolved within two weeks with only minor tissue destruction. In contrast, chronic skin inflammation results from a sustained, exaggerated inflammatory response, negatively affecting skin health.

### 3.1. Sunburn

Acute dermal overexposure to UV radiation causes sunburn and is an inflammatory response with increased prostaglandin and pro-inflammatory cytokine production, causing erythema, vasodilation, and leukocyte infiltration as predominant features [92,93].

Oxidative stress is thought to play a central role in the cellular response following UV exposure. Solar UV radiation is classified as UV-A (320–400 nm), UV-B (290–320 nm) and UV-C (100–290 nm) [94]. However, skin will only be exposed to UV-A and UV-B, as UV-C and partly UV-B radiation is absorbed by the ozone layer. UV-A photons are absorbed by endogenous UV-absorbing chromophores (e.g., riboflavin, quinines, tryptophan, and porphyrins) that subsequently transfer this energy to molecular oxygen, resulting in the formation of O_2_^−•^. Superoxide anions are then converted to H_2_O_2_ which in the presence of redox-active transition metals can be converted into HO• [95]. UV-B radiation is mainly absorbed by the epidermis and is primarily responsible for sun burn, whereas UV-A penetrates deeply into the skin. UV-A and UV-B radiation have different targets and outcomes. DNA is a prominent target of UV-B radiation, resulting in the formation pyrimidine and purine photoproducts and DNA strand breaks [96]. Additionally, UV-B-derived HO• can also damage the DNA, inhibiting normal cell function [97].

The effects of UV-A radiation exposure are a result of both direct and indirect damage to biomolecules and the subsequent physiological consequences hereof. Firstly, UV radiation damages skin lipids [98] and lipid peroxidation leads to increased production of prostaglandins, promoting inflammation in the skin [99]. Additionally, after UV exposure, keratinocytes and other skin-related cells upregulate pro-inflammatory cytokine production, e.g., IL-1, IL-6 and TNFα, and induce the expression of vascular adhesion molecules. TNFα is considered central to mediating UV-induced inflammation [100].

Furthermore, UV radiation of sunlight has been demonstrated to generate ROS leading to oxidative stress in skin due to depletion of endogenous antioxidant enzymes [101–103]. Chronically sun-exposed skin demonstrated no difference to sun-protected skin with respect to expression levels of Cu/Zn-dependent superoxide dehydrogenase (SOD)1, Mn-dependent SOD2, and catalase; however, sun exposure induced a marked increase in heme oxygenase expression [98]. ROS generated by UV radiation primarily cause damage to DNA through oxidative modifications and mutations, but also by inducing expression of different genes, such as matrix metalloproteinases and collagenases, thereby affecting collagen integrity and skin aging [104,105]. Additionally, UV-mediated ROS generation also indirectly affects cellular function and survival via its effect on cell signaling pathways [106]. For instance, activation of MAPK proteins occurs after UV exposure, suggesting that it may be responsible for executing the effects of UV-induced oxidative stress [93,98].

Thus, UV-induced oxidative damage is due to either immediate damage from UV radiation or indirectly from activated immune cells and dysregulated cellular signaling. An important aspect of UV damage is the depletion of the antioxidant defenses, which leaves the skin vulnerable to additional ROS damage [107]. This redox imbalance may also have systemic effects due to the wide-ranging effects of ROS, thereby rendering the body more prone to other ROS-mediated pathologies. This is further supported by several studies demonstrating an association between psoriasis and the prevalence and incidence of diabetes [108–110], a condition with ROS-mediated pathology due to increased ROS production and impaired antioxidant defense [111].

### 3.2. Psoriasis

Psoriasis is a chronic inflammatory skin disease manifested by red, thickened skin and skin scales due to keratinocyte hyperproliferation and is caused by a multitude of factors, including genetic, immunological, and environmental factors [112]. The pathogenesis of psoriasis includes complex interactions between skin and immune cells in concert with growth factors and pro-inflammatory chemo- and cytokines [113,114]; however, the exact underlying mechanisms still remain to be elucidated.

Oxidative stress is believed to be a key factor in the pathogenesis of psoriasis [115], as studies have suggested the involvement of increased ROS levels in psoriasis pathogenesis [101,116]. Increased ROS generation by infiltrated leukocytes into psoriatic lesions [117] is accompanied by substantial biomolecular damage, like psoriatic skin lesions containing oxidized LDL [118,119]. Indeed, a relationship between psoriasis severity, lipoprotein levels and oxidative damage has been proposed [120]. Notably, decreased antioxidant levels have been found together with increased levels of lipid peroxidation markers in blood of psoriasis patients [121,122]. Also, serum levels of catalase were elevated in psoriatic patients [123] and increased activity of superoxide dismutase (SOD) [124], and expression levels of peroxiredoxin (Prdx)2 and glutathione peroxidase (GPx)6 have been found in psoriatic skin lesions [125,126]. It is tempting to speculate that increased compensatory antioxidant levels counteract the skewed redox balance.

Besides direct damaging effects of unregulated ROS production, dysregulation of several pro-inflammatory pathways, like MAPK, NF-κB, and JAK-STAT, has been considered to contribute to psoriasis etiology [127]. Several members of the MAPK signaling pathways, like ERK1/2, JNK, and MAPK, were activated in psoriatic skin, further supporting this notion [128–130].

NF-κB, another redox-sensitive transcription involved in cellular processes like inflammation, cell proliferation and survival, has recently been demonstrated to be upregulated and active in psoriatic skin [129,131,132]. Dysregulation of NF-κB-mediated signaling may further exaggerate disease severity, as NF-κB-mediated upregulation of pro-inflammatory cytokines activates NF-κB via a positive feedback loop [133]. Importantly, inhibition of NF-κB nuclear translocation and DNA binding activity dampens the inflammatory component of psoriasis [134–136]. Many of the cytokines involved in psoriasis pathology induce keratinocyte proliferation and these signals are via ROS-mediated action transmitted to transcription factors and associated proliferation pathways [137,138]. Together, these observations strongly support a misbalanced oxidant/antioxidant balance as the major culprit in sustaining the inflammatory component in the pathology of psoriasis.

### 3.3. Burn Injury

A skin burn is a posttraumatic inflammatory condition accompanied by local as well as distant effects, leading to exaggerated inflammation, tissue damage, and infection. After skin burn, molecular signals, including inflammatory mediators and oxidants, are released at the injury site, further contributing to tissue damage and ischemic tissue necrosis [139,140]. Liquid from burn injury blisters contains a substantial amount of keratinocyte-derived pro-inflammatory cytokine IL-1β [141]. Moreover, induction of the inflammatory phase and attraction of immune cells leads to ROS production, exacerbating tissue damage [142]. Also, burns are often accompanied by secondary tissue damage distant to the site of injury [143–145] that is thought to be mediated by ROS and activated immune cells/neutrophils [146,147]. Besides local effects, burns also initiate systemic inflammatory reactions via ROS production, leading to distant oxidative damage to lipids and proteins [144,148]. Additionally, this systemic inflammatory response contributes to secondary damage as neutrophils have been demonstrated in various organs distant from the burn site within hours after injury and may lead to exaggerated oxidative stress and damage [145]. Notably, the intensity of the systemic inflammatory response and subsequently ROS generation correlates with the severity of the burn injury [149,150]. This pathological ROS production may affect cell signaling networks at the site of injury, thereby damaging cells and biomolecules. Also, ROS-mediated lipid peroxidation in skin is an important cause of cellular membrane dysfunction and subsequently cell death after burn injury [151,152]. A correlation has been described between the lipid peroxidation load and the degree of complications [153–156].

Importantly, these pathological effects seem to be caused by an overwhelming of the antioxidant systems, as decreased antioxidant scavenging capacity and reduced levels of SOD, GSH, and bilirubin have been reported after burns [148,156–159], likely due to massive consumption of these antioxidants as an attempt to counteract the oxidative stress. Thus, a tight control of the cellular redox balance is crucial, as excessive ROS production or decreased activity of ROS detoxifying enzymes results in aberrant wound healing [160] caused by exacerbated tissue damage due to macromolecular damage or by responses via stress-induced pathways.

## 4. Maintaining the Redox Balance in the Skin

**Figure 1:**
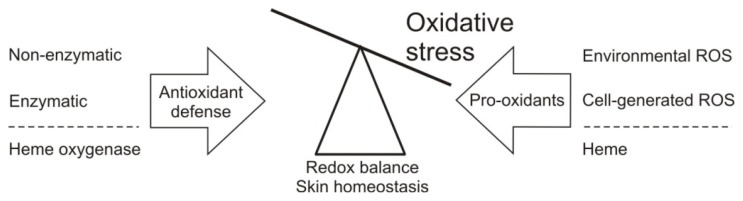
Redox balance maintenance in skin. ROS in the skin originate from normal cellular metabolism, e.g., mitochondrial respiration, and enzymatic activity. Besides, exogenous ROS are generated following physical insults, like UV light or persistent presence of leukocytes, facilitating chronic inflammatory skin conditions. To regulate ROS levels, the skin is rich in enzymatic and non-enzymatic antioxidant defense systems, thereby maintaining physiological homeostasis. In addition to the classical antioxidant defense, the cytoprotective enzyme heme oxygenase exhibits antioxidant properties via its degradation of pro-oxidant heme and generation of its antioxidant effector molecule bilirubin.

Free radicals are formed in the skin following exposure to environmental stimuli and immune reactions. In addition, ROS generation in skin occurs naturally as part of normal cellular metabolism, like mitochondrial respiration. These ROS are normally rapidly neutralized by non-enzymatic and enzymatic antioxidants, thereby maintaining the oxidant/antioxidant balance and thus tissue homeostasis [101] (Figure 1).

However, if the cytoprotective antioxidant factors get overwhelmed or are depleted, as may occur in hyperglycemic patients, the redox balance gets skewed towards oxidative stress, and may aggravate inflammatory injury.

### 4.1. Enzymatic Sources of Oxidants in the Skin

A major ROS source is the mitochondrial electron transport chain, leaking electrons during respiration. These electrons contribute to O_2_^•−^ generation that is released into both the mitochondrial intermembranal space and matrix [69]. Matrix O_2_^•−^ is converted to H_2_O_2_ by mitochondrial Mn-dependent SOD2 and can easily reach the cytosol by diffusion, whereas O_2_^•−^ from the intermembranal space exits the mitochondria via voltage-dependent anion channels and is converted to H_2_O_2_ by Cu/Zn-dependent SOD1 in the cytosol [161].

Various enzyme systems produce ROS secondary to their main enzymatic function, including xanthine oxidoreductase [162], lipid peroxidases [163], cytochrome P450 enzymes [164], and nitric oxide synthase [165]. Nitric oxide (NO) synthase produces the free radical NO, which, under normal conditions acts protective, but which under oxidative circumstances gets converted into peroxynitrite, which is highly damaging [166]. Moreover, under certain conditions, like loss of cofactor tetrahydrobiopterine, NO synthase uncouples and produces O_2_^•−^ instead of NO [167] leading to oxidative stress affecting cardiovascular performance [168].

The most important enzyme responsible for ROS production is the membrane–bound enzyme complex NAPDH oxidase (Nox). The Nox family consists of 7 members, Nox1-5 and Duox1-2, bearing a catalytic Nox or Duox domain, respectively. Furthermore, Nox isoforms contain a stabilizing domain (p22phox) and several regulatory subunits [169]. They display differential expression, regulation, and subcellular localization, and produce different ROS products [170]. Nox1, 2 and 5 are the key sources of O_2_^−•^, whereas Nox4 mainly produces H_2_O_2_ from molecular oxygen using NADPH as electron donor [171]. Nox-dependent ROS generation is activated by a wide range of chemical, physical, environmental, and biological factors [172]. The functions of the different Nox isoforms depend on their cellular localization and mode of activation [173] and have been thoroughly reviewed [174]. In keratinocytes, Nox1 is the only Nox expressed despite the detection of mRNAs encoding Nox1, Nox2, and Nox4 [175]. Keratinocyte Nox activity has been suggested to induce VEGF expression [176], MAPK activation [177], and cell growth [178]. Upon inflammation, infiltration of activated macrophages and neutrophils and their Nox2 and myeloperoxidase activities will exacerbate oxidative stress and prolong the inflammatory state [179].

### 4.2. Non-Enzymatic Sources of Oxidants in the Skin

Heme is crucial as the functional group of various hemoproteins, such as hemoglobin, cytochromes, peroxidases, and catalases [180]. In addition, heme can act as signaling molecule [181]. However, large amounts of free heme may be injurious to cells since heme is an iron chelate with the potential to catalyze iron-dependent reactions leading to ROS generation and membrane peroxidation [182]. Heme has indeed been demonstrated to catalyze ROS formation via the Fenton reaction [183] through its iron-dependent reaction with H_2_O_2_ [184,185].

After injury, large amounts of free heme are released from hemoproteins and aggravate tissue damage [182,186]. High local accumulation of heme can overwhelm the cellular ROS detoxification systems and prolongs oxidative and inflammatory stress [186–188]. Additionally, several studies have indicated that free heme also possesses pro-inflammatory properties [189–192], whereas low concentrations of free heme contribute to resolution of inflammation by downregulating inflammatory mediators, probably via induction of heme oxygenase (HO) activity [193–195]. Heme has therefore been suggested to act as a molecular switch due to its opposite, concentration-dependent effects [196].

Furthermore, free redox active metals, e.g., copper, zinc, and iron provide catalytic function to diverse (anti)oxidant enzymes [197], and redox cycling between Cu^+^/Cu^2+^ and Fe^2+^/Fe^3+^ is an integral part of the mitochondrial electron transport chain and ATP generation [198]. Transition metal homeostasis is regulated on both cellular and systemic levels and metal overload due to defective metal transporters is a central feature of several human diseases, like neurodegeneration [199]. Moreover, unregulated interaction of these metals with molecular oxygen also facilitates excessive ROS generation, predominantly via Fenton chemistry [200].

### 4.3. Antioxidant Systems in the Skin

Normally, the skin is constantly exposed to free radicals from the internal and external environment, challenging the functionality of the skin. Is our skin well enough prepared to withstand exogenous and endogenous ROS, and could targeting the redox status of the skin be a strategy to combat inflammatory skin conditions?

Under physiological conditions, ROS buildup in the skin is limited by numerous antioxidant defense systems, including both non-enzymatic and enzymatic mechanisms that either scavenge generated ROS before it can cause damage or prevent its formation (Figure 1, Table 1). In contrast to non-enzymatic antioxidants the enzymatic counterparts are not consumed and have a high affinity and reaction rate when scavenging ROS. Furthermore, the efficiency of the dietary non-enzymatic antioxidants depends on bioavailability as well as conversion into the active form upon ingestion [201] and antioxidant enzymes may therefore confer more efficient protection against acute oxidative and inflammatory stress [201].

The antioxidant defense system in skin is mainly comprised of the abundantly expressed antioxidant enzymes catalase, SOD, GPx, and Prdx [202].

SOD exists as three isoforms: cytosolic Cu/Zn-dependent SOD1, mitochondrial Mn-dependent SOD2, and extracellular SOD3 and catalyzes the conversion of O_2_^−•^ radicals into H_2_O_2_ using NAPDH as cofactor [203]. Excessive amounts of H_2_O_2_ are harmful to cells and rapid scavenging hereof is thus important [204]. This task is performed by either catalase, Prdx, or by GPx and the glutathione system that reduces the H_2_O_2_ to oxygen and water.

Mammalian cells may express 5 GPx isoforms [205], with GPx1 being the most prominent isoform in skin cells, reducing H_2_O_2_ and a range of organic peroxides [206]. GPx1 expression and activity is upregulated by ROS [207], and GPx1 and SOD display similar expression patterns [208], thereby securing an effective detoxification of H_2_O_2_ generated by SODs and avoiding the generation of HO• radicals. Moreover, GPx1 has been linked to the regulation of acute oxidative stress [209], as GPx1 knockout (KO) mice are highly susceptible to oxidative injury induced by paraquat and H_2_O_2_ [207] and GPx1 KO fibroblasts show increased sensitivity to oxidant-induced apoptosis [209].

Besides catalase and GPx, the six members of the Prdx family all catalyze the reduction of H_2_O_2_ and a wide spectrum of organic peroxides and peroxynitrite using GSH together with thioredoxin (Prdx1-5) or ascorbate (Prdx6), respectively [210]. Prdx6 is important in the cellular stress response, as Prdx6 overexpressing cells are protected from ROS-induced toxicity [211,212] and Prdx6 overexpressing keratinocytes are less sensitive towards photo-damage by UV radiation [213]. On the contrary, Prdx6 knockdown increases sensitivity towards oxidative stress [214–216].

Additionally, the NADPH/NADP+ ratio is an important index reflecting the cellular redox status. NADPH, the principal intracellular reductant, is generated from NADP+ by different groups of enzymes: (1) cytosolic glucose-6-phosphate dehydrogenase (G6PD) and 6-gluconate phosphate dehydrogenase; (2) cytosolic and mitochondrial isocitrate dehydrogenases; (3) cytosolic and mitochondrial malic enzymes; and (4) mitochondrial transhydrogenase [217]. NADPH is a central component in the cellular antioxidant system, as NADPH is required by glutathione reductase to reduce glutathione disulfide to GSH, an obligate co-substrate for GPxs [218]. Also, NAPDH is required by several pro-oxidant and antioxidant enzymes, including Nox and catalase [219]. G6PD is a major contributor to NADPH generation that despite its status as housekeeping enzyme is subjected to tissue-specific regulation by various factors, including oxidative stress, nutrients and hormones [220]. Notably, studies have shown that abrogation of G6PD activity dramatically increases cellular sensitivity to oxidative stress *in vitro* [221,222]. However, G6PD plays a dual role in the (anti)oxidative system as G6PD activity also provides Nox with NADPH. Obese and hyperglycemic rats demonstrated significantly higher Nox-derived O_2_^−•^ production due to increased G6PD activity and subsequently elevated NADPH levels to fuel this overproduction [223]. The resulting oxidative stress induced pathological changes of the heart and aorta and reduced cardiovascular function [223]. Together, this places G6PD and NAPDH centrally in the maintenance of a balanced ratio between pro- and antioxidants.

Besides the classical ROS-detoxifying enzymes discussed earlier, the HO system also exhibits potent antioxidant functions [224]. HO enzymes comprise the inducible HO-1 and the predominantly constitutively expressed HO-2 isoforms [225] and catalyze the degradation of heme into carbon monoxide (CO), iron, and biliverdin. Biliverdin is rapidly converted to bilirubin by biliverdin reductase [226,227]. Because heme is a redox active molecule that is cytotoxic in high concentrations [228], a direct beneficial effect of HO activity on the cellular redox balance can be ascribed to its active heme detoxification.

Numerous studies have linked the HO system to the regulation of various (patho)physiological processes, including cellular adaptation to oxidative stress and promotion of inflammatory resolution [229]. The expression of HO-1 is induced by various ROS-producing stresses [230], including heme and heavy metals [231], UV light [98,232], and H_2_O_2_ [233]. Also, oxidative stress-mediated induction of HIF-1 stimulates HO-1 activity in many tissues [234]. Actually, HO-1 induction is considered a marker of cellular oxidative stress and is involved in the protective response against oxidative damage [235].

Moreover, HO-1 overexpression counteracts the cytotoxic effects caused by high concentrations of free heme [181,190,236,237], whereas inhibition of HO activity intensifies oxidative cellular and tissue damage [238–241]. Preclinical and epidemiological evidence indicate that the cytoprotective and oxidative effects of the HO system are mediated via the generated effector molecules CO, bilirubin/biliverdin and by co-induced ferritin [For a recent review, see 230].

Both biliverdin and bilirubin generated from HO-mediated heme degradation are strong antioxidants [242,243], and bilirubin ameliorates oxidative stress in different diseases, including atherosclerosis, diabetes mellitus, and inflammatory and autoimmune diseases (Recently reviewed in [244]). For instance, bilirubin protects cells against high H_2_O_2_ concentrations [242,245,246] and nanomolar concentrations of bilirubin suppress Nox activity *in vitro*, thereby reducing ROS levels and subsequent tissue damage [247–249]. Thus, the involvement of biliverdin and bilirubin in antioxidant defense is evident [250].

Ferrous iron (Fe^2+^) is released during heme degradation by HO enzymes and can participate in ROS-generating Fenton chemistry, leading to cellular damage [251]. Ferritin is a ubiquitous iron-binding protein that can accommodate up to 4,500 iron atoms per molecule [252,253]. Notably, ferritin expression is not only regulated by cellular iron levels but also by oxidative stress via Nrf2 binding to an antioxidant responsive element (ARE) in the ferritin promoter [254], leading to co-induction with HO-1 [196]. Thus, the HO system contributes significantly to the cellular antioxidant defense by degrading redox active heme, thereby generating ROS-targeting effector molecules that further contributing to counteracting oxidative stress.

Numerous studies using cellular and animal models have demonstrated protective effects of vitamin C, a water-soluble compound, and vitamin E, a fat-soluble compound, against cytoplasmic oxidative damage and lipid peroxidation, respectively, in the etiology of atherosclerosis. Disappointingly, clinical studies have turned out less promising [255].

Together, under normal physiological conditions, ROS-mediated reactions in the skin are well balanced and protect the cells against oxidative stress. However, under pathological conditions like inflammation excessive damaging ROS formation may occur due to overwhelming of the antioxidant systems, contributing to a worsened clinical outcome.

## 5. Oxidative Stress as Therapeutic Target in Inflammatory Skin Conditions

The skin is constantly subjected to ROS formation via UV irradiation, environmental exposure, and cellular metabolism and is rich in enzymatic and non-enzymatic antioxidant systems keeping the ROS levels at homeostatic levels. However, inflammatory conditions in combination with an overwhelmed antioxidant system leads to pathological levels of ROS and oxidative stress, further exacerbating disease state. Attenuation of ROS levels and restoring the redox balance could then normalize the inflammatory response.

A potential therapeutic approach to restore antioxidant levels in the skin could therefore be achieved through (1) reducing the ROS production; (2) increasing endogenous antioxidant enzymatic defenses; or (3) enhancing the non-enzymatic antioxidant defenses via dietary or pharmacological approaches.

### 5.1. Reduction of ROS Production

#### 5.1.1. Nox Inhibition

Increased ROS production and oxidative stress contribute to cellular and tissue injury during the progress to chronic inflammatory skin diseases. Recent studies suggest that Nox-generated ROS contribute to cellular and tissue damage by fuelling the acute inflammatory response [174,256–259]. For instance, studies indicate that Nox-generated ROS contribute to TNFα-mediated activation of NF-κB and vascular adhesion molecule expression [256,260–262]. Indeed, the central role of Nox in a multitude of diseases suggests it to be a putative therapeutic target, and several Nox-targeting inhibitors have been developed [263,264] with the main focus on the macrophage-specific Nox2 due to its involvement in several inflammatory conditions [173]. However, a recent study demonstrated that genetic Nox deficiency enhanced inflammatory responses after LPS challenge *in vivo*, suggesting that Nox-generated ROS in certain settings also have anti-inflammatory functions [265,266]. These contradictive and highly condition-specific outcomes of Nox-generated ROS may in the future challenge the use of Nox as therapeutic target to counteract redox imbalances.

#### 5.1.2. Metal Scavenging Proteins

Redox active metals obstruct cellular signaling pathways by ROS-dependent and independent mechanisms. Both enzymatic and non-enzymatic antioxidants protect against metal-mediated ROS generation by (i) chelating redox-active metals; (ii) maintaining the metal redox state and preventing Fenton chemistry; and (iii) scavenging of metal-mediated ROS [200].

Excess free iron released after insults can catalyze ROS formation via Fenton reaction and cause tissue damage [228]. Furthermore, under *in vivo* stress conditions, an excess of superoxide releases iron from iron-binding molecules, including ferritin [267], contributing to further iron overload that can have deleterious effects [268,269]. Thus, the application of suitable metal chelators may contribute to a reduction in metal-induced ROS formation.

Chelation therapy is medical treatment for heavy metal poisoning and scavenging of redox active metals. Normally, labile cellular iron not contained by ferroproteins is scavenged by ferritin, thereby neutralizing the pro-inflammatory and pro-oxidative potential of free iron and preventing immune-mediated inflammatory conditions [270]. The bacterial siderophore Deferoxamine is an iron chelator frequently used to treat acute iron overload and related complications [271]. Also, plant-derived polyphenolic compounds (so-called botanicals) can be effective metal chelators [272]. For instance, the antioxidative effect of the plant-derived flavanoid quercetin is predominantly due to its chelating of redox-active iron [273].

### 5.2. Increasing Enzymatic Antioxidant Defenses

#### 5.2.1. Antioxidant Upregulation

As mentioned earlier, superoxide scavenging is performed by the three mammalian SOD enzymes. The primary location of SOD3 is the extracellular matrix and on cell surfaces, whereas SOD1 and SOD2 are intracellularly located [274]. SOD3 is expressed in the epidermis and dermis of skin [275], but the role of SOD3 in this tissue is less clear. SODs have been suggested to be involved in the defense against UV-mediated ROS, as SOD enzyme expression and activity are affected by UV exposure [276–278]; however, SOD3 needs significantly higher UV doses before being activated than SOD1 and SOD2.

Numerous studies have investigated the antioxidative effects of elevated SOD levels in tissues by different means. For instance, intramuscular injections of SOD1 were successfully applied as anti-fibrotic therapy in treating cutaneous radiation-induced fibrosis in humans [279] and similar promising results were obtained with SOD2 in a porcine model of radiation-induced fibrosis [280].

Additionally, cutaneous SOD2 gene therapy reduced superoxide levels and normalized wound healing in mice with chemically-induced diabetes [281]. Similarly, diabetic transgenic SOD2 mice also demonstrated reduced superoxide levels and improved wound healing after ischemic stress compared to wild type controls [282].

Notably, chemically-induced contact dermatitis was alleviated in transgenic mice overexpressing SOD3 under the control of the keratin14 promoter, including a reduction in the levels of ROS and pro-inflammatory cytokines as well a reduced immune cell infiltration [283,284]. By contrast, SOD3 KO mice display exaggerated IL23-mediated psoriasis-like skin inflammation, including increased immune cell infiltration and higher levels of pro-inflammatory cytokines compared to WT controls [285,286], suggesting a role for SOD3 in cutaneous inflammation. Also, SOD3 expression is reduced in psoriasis patients compared to healthy subjects [284], further supporting this.

Moreover, SOD3 is suggested to play a role in pulmonary, arthritic, and neurological conditions [287]. Importantly, increasing SOD3 levels in various experimental disease models, e.g., chemically induced diabetes, hypertension, and inflammatory arthritis, reduced oxidative stress and improved disease state [288], thereby placing SOD3 as a central therapeutic target. Many studies have focused on SOD3 therapy in targeting ROS-mediated cardiovascular effects; however, the outcome has been variable [288]. Importantly, this discrepancy may relate to the use of predominantly rats in these studies despite that fact that rat SOD3 differs structurally and chemically from other mammalian SOD3 forms, resulting in a lower SOD3 concentration in rat vascular tissues [289], probably creating a therapeutic window that would not be present in other mammals, e.g., dogs, that did not show a therapeutic benefit from SOD3 gene therapy [290].

Thus, it is clear that increasing the enzymatic antioxidant defense by exogenous applications like injection or gene transfer may be a future therapeutic approach for multiple disorders with an inflammatory component; however, more research in terms of tissue- and condition-specific effects is warranted about the role of the SOD enzymes in skin inflammation.

#### 5.2.2. Synthetic Antioxidants

Low-molecular mass synthetic compounds exhibiting catalytic activity, thus operating as enzyme mimics, have been developed as putative antioxidant therapy. The first mimetics were SOD-like because SOD is the first line of antioxidant defense, and today different classes of SOD mimetics based on metalloporphyrins, manganese (Mn) cyclic polyamines, and salen Mn derivatives have been developed [291]. Fortunately, their chemical and biophysical properties not only make them potent SOD mimics but also allow them to neutralize other types of ROS, including peroxynitrite and H_2_O_2_, and can thus also be considered catalase/peroxidase mimetics [292]. This broad specificity allows for modulation of the cellular redox environment and thus confers advantages over non-enzymatic antioxidants [293].

Moreover, these compounds have been shown to be effective in reducing oxidative stress in different *in vitro* cytotoxicity models involving ROS production [293,294] as well as in numerous *in vivo* models [292]. Notably, many of these compounds are not only functionally protective but also reduce oxidative damage to biomolecules [295–300] and the salen Mn compounds confer protection against mitochondrial damage [299].

Importantly, these mimetics display anti-inflammatory potential as H_2_O_2_ mediates activation of inflammatory genes as mentioned earlier, and therefore conditions with an inflammatory component may be the main target for such strategy. Also, reduced levels of activated macrophages were reported following treatment with mimetics in a radiation-induced lung injury model, however, no effects on pro-inflammatory cytokine levels were detected [300]. Also, a recent study demonstrated beneficial effects of systemic mimetic treatment on wound healing after skin irradiation by reducing oxidative damage [301].

Thus, these compounds do show promising prospects for therapeutic strategies in treating ROS-mediated complications in a wide range of conditions. Unfortunately, comparative studies on the different synthetic antioxidant classes in *in vitro* and *in vivo* settings are still very sparse and so far none of the antioxidant mimetics has been approved for clinical use.

#### 5.2.3. Induction of the HO System

HO-1 is a stress-responsive enzyme with both antioxidant and cytoprotective effects mediated by the generated effector molecules biliverdin/bilirubin and CO, placing HO-1 as a central player in protection and homeostatic re-establishment after a wide range of pathological insults [302]. Numerous studies have demonstrated significant therapeutic effects of upregulation of HO-1 expression and/or activity as well as effector molecule administration in multiple pathological inflammatory conditions [230,302]. Also, ferritin is co-induced with HO-1 induction, which may provide a beneficial side effect, as ferritin scavenges free iron and thereby contributes to a restoration of the redox balance.

Recently, strategies to employ HO-1 as therapeutic target have been considered. This is further substantiated by HO-1 gene deficiency cases [303,304] and by the fact that promoter polymorphisms of the HMOX1 gene affect HO-1 protein expression levels and, subsequently, the severity of pathological human conditions [305]. Being an inducible enzyme, several synthetic molecules, including porphyrins [306] and heme arginate [307] have been developed and identified as possible HO-1 inducers. Heme arginate has for years been used as a clinical approach to treat porphyria, a disorder caused by non-functional heme metabolism [308]. Lately, a focus has been shed on the expanding number of plant-derived dietary compounds, so-called phytochemicals, with HO-1 inducing effects [309], like curcumin [310–312] or quercetin [313,314]. However, the effects of these pharmacological non-toxic, low-cost compounds still need to be studied in more detail before clinical use.

### 5.3. Increasing Non-Enzymatic Antioxidant Defenses

#### 5.3.1. Oral and Topical Administration

Epidemiological studies have suggested that consumption of antioxidant-rich food is associated with lower disease rates and preventive protection of cardiovascular disease [315]. Thus, the supplementation of non-enzymatic, dietary antioxidants could be a feasible way of restoring redox homeostasis and reduce ROS-associated diseases.

However, clinical studies employing antioxidant supplement therapy have been inconclusive. For instance, the antioxidant compound *N*-acetyl cysteine (NAC) has been successfully used in the treatment of idiopathic pulmonary fibrosis [316–319], an inflammatory condition with etiology linked to Nox4-mediated ROS generation [320]; however, this therapeutic effect may not purely be ascribed to direct antioxidant effects of NAC itself but to its link to cellular glutathione replenishment [321]. Also, the combined use of NAC together with other commonly used treatment protocols should be carefully dissected and considered for each patient situation, as a recent study demonstrated increased mortality and severe treatment-related adverse effects when employing NAC in a three-drug regimen [322].

Frequently studied dietary and naturally occurring antioxidants such as carotenoids, flavonoids, and several vitamins have been implicated as promoters of skin health and rejuvenation [323]. External factors like chronic sun exposure, smoking, and pollution are significant contributors to skin aging, and both vitamins C and E have been demonstrated to have differential UVB photoprotective effects when applied both topically and orally [324,325]. Moreover, oral combination therapies of vitamins C and E resulted in a dramatically increased photoprotective effect compared to monotherapies [326]. Also oral intake of β-carotene or provitamin A reduces UV-induced erythema formation in different clinical studies; however, this effect is highly dose- and time-dependent [327–329]. Notably, β-carotene has been demonstrated *in vitro* to quench UV-induced radical formation and lipid peroxidation [330,331] and to reduce mitochondrial mutagenesis after UV exposure of skin fibroblasts [332].

Polyphenols are plant-derived micronutrients such as green tea polyphenols and curcumin that also have gained more attention in skin research during the last decade due to their antioxidant properties and their potential beneficial effects on cancer, neurodegenerative and cardiovascular diseases that all have been linked to oxidative stress [333]. Various polyphenols have been reported to be photoprotectors [334]. Oral as well as topical application of green tea polyphenols to mice protected in a time-dependent manner against UV-induced cutaneous edema, depletion of the epidermal antioxidant defense, and cyclooxygenase induction [335,336]. Studies using animal models have also demonstrated anti-inflammatory activities of administration of green tea polyphenols, predominantly mediated by the major green tea component epigallocatechin-3-gallate [337,338]. Also in humans topical application of green tea polyphenols dose-dependently reduced erythema formation and sunburn cells [337]. Sunburn cells are keratinocytes undergoing apoptosis due to irreversible DNA damage [339]. However, the underlying mechanism of these compounds is still not well understood but has been suggested to be mediated via effects on signal transduction pathways [340].

Another powerful antioxidant involved in skin antioxidant defense is coenzyme Q10 (CoQ10) [341]. However, being part of the respiratory chain, CoQ10 also contributes to ROS formation, as discussed earlier. In skin, the CoQ10 level is 10 times higher in the epidermis compared to the dermis [341]. Being the outermost skin layer, the epidermis is directly exposed to UV irradiation and it is known that UV irradiation depletes antioxidants in the skin [107]. The epidermis may thus be an optimal target for CoQ10 administration. Dietary CoQ10 supplementation to rats increased the levels of CoQ10 and its homologs in tissues and mitochondria therein [342]. This was accompanied by a reductive shift in plasma aminothiol status and decreased oxidative damage to mitochondrial proteins in skeletal muscle [342]. In contrast, mice on CoQ10 enriched diets did not show any effect on the systemic redox balance, nor the lifespan, despite a buildup of CoQ10 in tissues [343]. Notably, human epidermal keratinocytes isolated from topically CoQ10-treated skin demonstrated improved mitochondrial function and protection against UV-induced mitochondrial damage compared to non-treated controls [344]. Other studies demonstrated that CoQ10 stabilizes mitochondrial function, improves cell viability, and attenuates oxidative effects in human skin [345,346].

In summary, despite their great availability and use, the effects of dietary supplements on skin and general health still remain controversial. However, several things that could be crucial for treatment outcome must be considered. Firstly, these bioactive compounds have to be prepared and taken up by the target organ. The stability of these compounds will ultimately determine efficiency, and the use of vitamin C in creams has proven difficult due to a low stability in the presence of oxygen [347]. To account for this and to facilitate uptake, more stable derivates are often used though several of these compounds are not efficiently converted into the active form of the antioxidant [348]. Another issue is the bioavailability of the oral supplements. Dietary supplements have to pass through the gastrointestinal tract, enter circulation, and reach the target tissue and selected cell types and/or cellular compartments, which may be important for the effective dose to be given. Also, toxic effects due to e.g., cross-reactions and organ-specific reactions to certain compounds should be taken into account [349]. Additionally, route of administration is important, as e.g., curcumin is effective to skin when applied topically, but only to the colon when applied orally [340]. Importantly, antioxidant supplementation may interfere with the endogenous antioxidant response normally initiated after exercise and subsequently interferes with ROS signaling [350]. Other clinical studies on vitamins A, C and E, coenzyme Q10, carotene, and plant-derived flavanoids have turned out rather disappointing, as no significant effects these dietary supplements on general health have been detected [351,352]. More importantly, the long-term effects of many of these supplements are yet to be assessed, but one study linked increased mortality to long-term intake of antioxidant supplements [353].

Interestingly, the oxidated targeted site may determine the success of antioxidant therapy. Although both GSH and bilirubin are potent endogenous antioxidants, they protect against distinct targets. GSH mainly protects against hydrophilic proteins, whereas bilirubin protects against lipid peroxidation [354]. Recently, we demonstrated that iatrogenic induction of mild hyperbilirubinemia ameliorated the serum antioxidant status and vascular function in diabetic patients [355]. Thus, more in-depth studies are still needed to gain more knowledge about *in vivo* and *in vitro* obtained data on the diverse group of potential beneficial antioxidants. Knowledge concerning dose-response, optimal administration, cellular and compartmental targeting and translational studies are still urgently needed.

#### 5.3.2. Targeted Antioxidant Delivery

Mitochondria are central organelles in cell survival and function [356] and increasing attention has been paid to the role of mitochondrial dysfunction in aging, apoptosis, neurodegeneration, and cancer [357]. Despite already being equipped with antioxidant systems, great interest has been paid to developing supplementary approaches to further protect the mitochondria from ROS-mediated damage. For instance, CoQ10 and related ubiquinones have been used as therapy to decrease mitochondrial damage in Parkinson’s disease patients [358,359]. However, delivery issues limited the therapeutic effect as oral administration only resulted in a limited mitochondrial uptake of ubiquinones [360–362]. Conjugation of a lipophilic triphenylphosphonium cation to ubiquinones led to the development of MitoQ, which selectively is taken up by and accumulated within mitochondria [363]. Moreover, MitoQ efficiently prevented oxidative stress in isolated mitochondria, as well as in the ischemic heart [364–366]. Later, other effective compounds such as SkQ1 and Trolex were developed and characterized [367–369]. Lately, nanotechnology has been employed in targeted mitochondrial delivery. Different drugs employed in cancer, Alzheimer’s disease, and obesity demonstrated improvement in the drug therapeutic index after nanoparticle-mediated delivery compared to a non-targeted carrier or to the free form of the therapeutics [370].

Notably, a recent report described induction and subsequently mitochondrial translocation of HO-1 following gastric mucosal injury, resulting in prevention of mitochondrial oxidative stress and pathology [371], suggesting that HO-1 induction may represent another mitochondrial targeting strategy. Together, this targeted approach thus holds big potential for future treatment strategies.

## 4. Concluding Remarks

ROS are crucial for cellular functions and provide essential protective mechanisms. However, ROS and oxidative stress have also been linked to various disease states, including inflammation, diabetes mellitus, cardiovascular diseases, and aging [88]. A well-balanced redox homeostasis is important, as oxidative stress either by increased levels of ROS and/or by depleted antioxidant systems may dysregulate protein function due to oxidative modifications and further aggravate inflammatory conditions. Targeting oxidative stress in inflammatory skin conditions may ameliorate disease outcome by dampening inflammation and improving recovery. However, although promising, efficacy and clinical studies employing antioxidant therapy are still in its infancy.

## Figures and Tables

**Table 1:** Enzymes and factors involved in antioxidant defense in the skin.

Antioxidants	Examples	Target
Enzymatic	Superoxide dismutase	Superoxide
	Catalase	Hydrogen peroxide
	Glutathione peroxidase	Hydrogen peroxide, lipid peroxides
	Peroxiredoxin	Hydrogen peroxide
	Heme oxygenase	Heme

Non-enzymatic	Bilirubin	Lipid peroxides
	Vitamin C	Superoxide, hydroxyl radical, reactive nitrogen species, trace metals
	Vitamin E	Lipid peroxides

Metal-binding proteins	Ferritin	Free iron

## Abbreviations

APE/Ref-1: apurinic/apyrimidinic endonuclease/redox effector factor-1
ARE: antioxidant responsive element
CO: carbon monoxide
CoQ10: coenzyme Q10
ERK: extracellular signal-regulated protein kinases
G6PD: glucose-6-phosphate dehydrogenase
GPx: glutathione peroxidase
GSH: reduced glutathione
H_2_O_2_: hydrogen peroxide
HO•: hydroxyl radical
HIF-1: hypoxia inducible factor-1
HO: heme oxygenase
HRE: hypoxia response element
JNK: c-Jun *N*-terminal kinases
KO: knockout
LDL: low density lipoprotein
MAPK: mitogen-activated protein kinase
Mn: manganese
NAC: *N*-acetyl cysteine
NF-κB: nuclear factor-κB
Nox: NADPH oxidase
Nrf2: NF-E2-related factor 2
O_2_^−•^: superoxide anion
Prdx: peroxiredoxin
PTP: protein tyrosine phosphatase
ROS: reactive oxygen species
SOD: superoxide dismutase.
